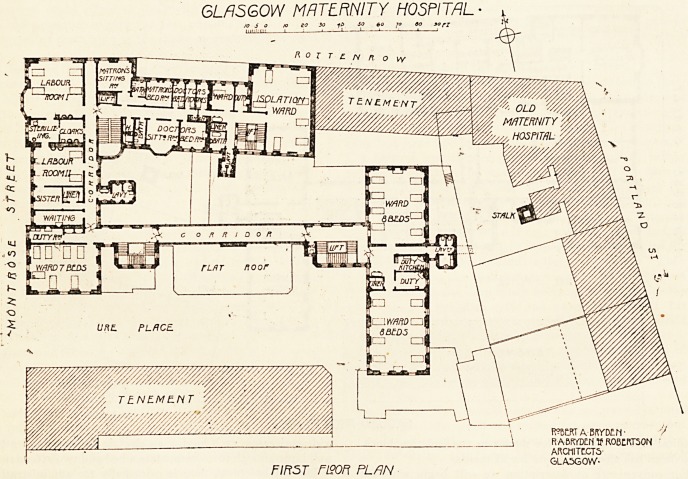# Glasgow Maternity and Women's Hospital

**Published:** 1908-05-09

**Authors:** 


### GLASGOW MATERNITY AND WOMEN'S HOSPITAL.

The New Hospital, which was formally opened on Wednesday, April 29, by Her Grace the Duchess of Montrose, is situated on a commanding eminence immediately alongside the Old Maternity Hospital, which in future will be used for the treatment of gynæcological cases.

The site measures 250 ft. long by 207 ft. broad, and owing to the natural fall in the ground gives two flats in the southern wing below the main entrance floor on the level of Rottenrow. In some respects the site presented difficulties, as the ground southwards falls very suddenly, thus necessitating the erection of substantial retaining walls to carry the buildings on the higher level.

The hospital provides accommodation for 108 patients—92 in the general maternity department and 16 in the isolation block. There are 44 bedrooms for nurses in the general block. The admission department has been designed so that an unsuitable case is at once detected, and cannot gain admission to the general maternity wards. To the left of the entrance hall there is a board-room, cloak-room, and lavatory, matron's office and district nurse's room, with a stair and electric lift leading to the isolation wards. In the south-west corner of the building there is a large lecture theatre occupying two stories in height, and giving accommodation for 90 students. The patient and lecturer enter the lecture hall from the sub-basement level, and the students enter from the level of the ground floor into the gallery. On this flat are five wards containing from four to eight beds, with the necessary lavatory accommodation, ward kitchens, etc. There is also the residents' dining-room, dispensary, a room for the visiting staff, and three

GLASGOW MATERNITY HOSPITAL·

GROUND FLOOR PLAN·

hospital and 4 bedrooms for nurses in the isolation block. Provision has also been made for 21 maids.

For convenience of description we will begin with the ground floor plan, of which we give an illustration.

The main entrance to the hospital is on this level. There is a covered carriage-way, which permits patients being removed from the ambulance without being observed from the street. Adjoining the vestibule is the porter's room, inquiry office, and telephone exchange. Immediately to the right is the admission department, and on the left is a free air space cutting off the isolation block. When a patient arrives she is immediately taken into the waiting-room, where the necessary particulars regarding her are ascertained. She is then examined in the preliminary examination room, and if found a suitable case for admission to the general maternity wards, is taken to the undressing room, situated on the opposite side of the corridor, then to the bathing-room, and thereafter to the examination-room proper. Should it be found in the preliminary examination-room that the patient is not suitable for the general maternity wards, she is immediately transferred to the isolation wide staircases, each having an electric lift running up the well large enough to accommodate a bed and two attendants.

The sub-basement floor is principally for administrative purposes, and here are the nurses' dining-room, sitting-room, and bedrooms. Below the entrance hall of the admission department is the museum, private laboratory, and in a detached building is the mortuary, post-mortem room, and small chapel where funeral services may be held.

In the basement is a large well-equipped kitchen, communicating with the nurses' dining-room through a servery by an electric lift. There is also accommodation for maids and a servants' dining-hall, as well as the laundry, washing-house, boilers, disinfecting chamber, etc. A special feature in the laundry department is the precaution which has been taken for the treatment of all linen coming from the isolation block. The linen from this department is first of all steeped in tubs in a small washhouse, and then boiled before being passed through to the general washhouse. The entrance to this isolation washhouse is quite separate from the entrance to the general washhouse, so that there is scarcely any possibility of the linen getting mixed.

On the first floor (the plan of which we publish) in the centre of the north block, and reached by a private corridor, are placed the resident doctors' sitting-room and three bedrooms with bath-room. To the west of these are the matron's apartments, consisting of sitting-room, bedroom, and bathroom, all entering from a private lobby. On the north-west corner of the building are situated two labour rooms—a large one for four beds and a smaller one for two beds. These apartments have a splendid north-west light, the walls are tiled to the ceiling, and the floors are constructed in terrazzo. Adjoining these labour rooms, which are equipped on the lines of a modern operating theatre, are a sterilising room, robing-room with lavatory, waiting-room for students, and duty-room for the sister-in-charge. On the south-west corner of the building on this level is a ward for seven beds, and a corridor leading to the east block, which is glazed on both sides, where there are two wards, each containing eight beds, with lavatory accommodation, nurses' duty-room, and ward kitchen.

building. The first floor of the isolation block is occupied by a ward of seven beds, and a small ward to accommodate a single bed, also sleeping accommodation for the resident doctor. The second floor provides for eight patients, while on the top flat there is a well-equipped operating-room fitted with a large north light and an anæsthetic room adjoining. Entered from a private corridor are four bedrooms for the nurses employed in this department.

Generally the interior of the building has been finished in the simplest manner possible commensurate with hospital requirements of the present day. The corridors throughout the building and the main floors are laid with grey terrazzo, and the basement with granolithic. The walls of the corridors are tiled and painted white. The ward floors are of fire-resisting concrete, covered with maple flooring. The lavatory fittings are of the most up-to-date pattern.

The heating is effected by means of hot water circulated at low pressure on the "Reck" system, and by its rapid

GLASGOW MATERNITY HOSPITAL.

FIRST FLOOR PLAN.

The second floor plan contains wards of various sizes ranging from two to eight beds, and, on the south-east corner, bedrooms for nurses. There is a special room for the bathing of babies. This is a well-lit apartment: the floor is formed with terrazzo, the walls are tiled, and the room is specially heated by radiators, as well as an open fire. Round it are hot and cold water taps to supply small portable baths. Here the nurse can conveniently bathe the children, and in this way prevent them disturbing the mothers in the wards.

The third floor plan in the main building contains a modern operating theatre with anæsthetic room, sterilising room, room for preparing dressings, and robing-room. In addition the remainder of the nurses' bedrooms are arranged in the south-east corner of the building.

The isolation block, which occupies the north-east corner of the site, is completely cut off from the hospital proper by a ventilating corridor on the ground floor, the upper flats having no communication whatever with the rest of the movement through the pipes and radiators it is easy to preserve an equable temperature in either summer or winter. The building is lit by electricity, and inter-communicating telephones have been fitted throughout.

The total cost of the site was £15,000, and of the buildings, £60,000—£75,000 in all.

The building was designed by Mr. R. A. Bryden, F.R.I.B.A., and until his death, two years ago, its erection was superintended by him. Since then the building has been superintended by Mr. Robertson, of the firm of R. A. Bryden and Robertson, architects. Before the plans were completed the directors commissioned Dr. D. J. Mackintosh, Superintendent of the Western Infirmary, who has acted as expert adviser, along with two members of the visiting staff, Drs. Jardine and Munro Kerr, and their architect, Mr. Bryden, to visit the principal maternity and gynæcological hospitals at home and on the Continent, in order that the new institution might embody the latest and best features for the treatment of maternity cases. The deputation visited all the leading hospitals in this country as well as those on the Continent, and upon the experience thus gained the new hospital has been designed and equipped.

## Figures and Tables

**Figure f1:**
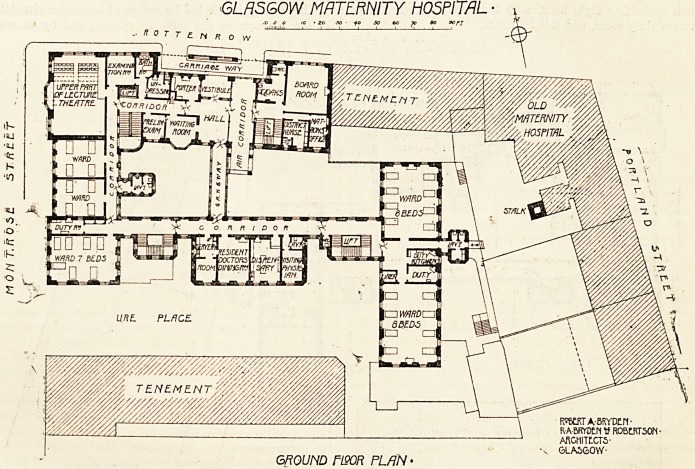


**Figure f2:**